# Decoding Sebaceous Gland Biology Through Experimental Models: Progress, Limitations, and Future Directions

**DOI:** 10.3390/cells15171591

**Published:** 2026-09-01

**Authors:** Stefania Briganti, Sarah Mosca, Monica Ottaviani, Enrica Flori

**Affiliations:** Laboratory of Cutaneous Physiopathology and Integrated Center of Metabolomics Research, San Gallicano Dermatological Institute, IRCCS, 00144 Rome, Italy; sarah.mosca@ifo.it (S.M.); enrica.flori@ifo.it (E.F.)

**Keywords:** sebaceous gland, sebocyte biology, 3D skin models

## Abstract

The sebaceous gland (SG) is a highly dynamic skin appendage that plays a central role in maintaining skin homeostasis through lipid production, immune modulation, and interactions with the skin microbiome. Dysregulation of SG function is implicated in several dermatological disorders, including acne and other inflammatory skin diseases. Over the past decades, a wide range of experimental models has been developed to investigate SG biology and pathology. These include in vivo animal models, ex vivo human skin explants, primary sebocyte cultures, and immortalized sebocyte cell lines, each offering distinct advantages and limitations. More recently, significant progress has been made in the development of advanced three-dimensional (3D) models of sebaceous gland biology, including sebocyte spheroids, SG organoids, human skin equivalents containing sebaceous components, and organotypic co-culture models, which better recapitulate the structural and functional complexity of SGs. In particular, the emergence of SG organoids derived from adult or pluripotent stem cells represents a breakthrough, enabling the study of sebocyte differentiation, lipid metabolism, and cell–cell interactions in a physiologically relevant context. Despite these advances, current models still face important challenges, including incomplete cellular maturation, limited representation of the immune and vascular components, and insufficient modeling of the native skin microenvironment. Future developments integrating bioengineering approaches, microfluidic platforms, and multi-cellular systems are expected to further enhance model complexity and translational relevance. This review summarizes current knowledge on experimental models developed to study SG and sebocyte physiology and pathology.

## 1. Introduction

Sebaceous glands (SGs) are specialized exocrine structures that constitute a key component of the pilosebaceous unit, contributing to skin homeostasis and barrier integrity. They are widely distributed across the human body, with the highest density in seborrheic regions such as the face, scalp, chest, and upper back, while being absent on the palms and soles [1,2]. SGs originate during embryogenesis as outgrowths of the hair follicle epithelium and share a common stem cell lineage with both the interfollicular epidermis and hair follicle [3,4]. Anatomically, they are multi-lobular holocrine glands located in the dermis and typically connected to the upper portion of the hair follicle via a ductal structure [1,4]. Each gland is composed of acini containing sebocytes at different stages of differentiation, arranged from a peripheral proliferative layer to a central zone of fully differentiated cells. Sebocytes undergo a tightly regulated differentiation program characterized by progressive lipid accumulation, ultimately culminating in holocrine secretion, in which mature cells disintegrate and release their contents into the follicular canal [5,6,7,8,9].

The primary product of SGs, sebum, is a complex lipid mixture composed mainly of triglycerides, wax esters, squalene, cholesterol, and free fatty acids [10,11]. Notably, certain components such as squalene and wax esters are uniquely synthesized by sebocytes [12,13]. Sebum plays multiple physiological roles, including lubrication of the skin and hair, maintenance of barrier function, regulation of surface pH, and protection against environmental stressors [12,13,14,15]. In addition, it contributes to antimicrobial defense and antioxidant activity at the skin surface [1,7].

Sebaceous gland activity is tightly regulated by systemic and local factors, particularly hormones. Androgens, especially dihydrotestosterone, are the drivers of sebocyte proliferation and lipid synthesis, while other endocrine signals such as estrogens, glucocorticoids, and insulin-like growth factor-1 further modulate gland function [12,16,17]. Consistent with this regulation, sebum production varies across the lifespan, increasing markedly during puberty and declining with age [6]. Beyond endocrine control, sebocytes express a wide range of receptors, including those for neuropeptides (such as corticotrophin-releasing hormone (CRH) receptor and melanocortin receptors (MC1-5R) and lipid-sensing nuclear receptors (e.g., peroxisome proliferator-activated receptors (PPARs)), enabling them to integrate environmental and metabolic cues [1,18,19,20].

In addition to their secretory function, SGs are increasingly recognized as active participants in cutaneous immunobiology. Sebocytes can produce cytokines, chemokines, and antimicrobial peptides, and respond to innate immune stimuli, thereby contributing to local immune surveillance. Moreover, sebum-derived lipids can directly modulate microbial growth and immune responses [21,22,23,24,25]. These features, together with their interactions with keratinocytes, fibroblasts, and immune cells, position SGs as important regulators of the skin microenvironment [26,27,28,29,30].

Given their complex structure, unique differentiation program, and multifaceted functional roles, SGs represent a biologically dynamic system. These features make them particularly relevant for the development of in vitro and in vivo models aimed at investigating skin homeostasis, disease pathogenesis, lipid metabolism, host–microbe interactions, and tissue-specific immune responses. Importantly, the ability of an experimental model to recapitulate key aspects of sebocyte biology is essential for assessing its biological relevance. Accordingly, changes in sebocyte differentiation, lipid synthesis, and intracellular lipid accumulation are widely used as molecular and phenotypic parameters for the characterization and validation of SG models. The principal molecular and phenotypic parameters used to assess these features are summarized in Table 1. This review provides a comprehensive overview of currently available experimental models for SG research, highlighting their applications, limitations, and potential for advancing our understanding of SG biology and associated diseases.

## 2. Material and Methods

For this narrative review, we performed a comprehensive PubMed literature search for relevant reviews and studies reporting original experimental data involving human or animal SGs, primary sebocytes, sebocyte cell lines, sebocyte-based co-culture systems, 3D SG-like structures, SG-containing skin models, organoid systems, or bioengineered and microfluidic models involving SGs or sebocytes. Reference lists of relevant articles and review papers were also manually screened to identify additional publications that were not retrieved through the electronic database search. Searches were conducted for all English peer-reviewed articles, with a preference for more recent literature. Our search was performed from 15 April 2026 to 20 August 2026. The search strategy combined terms related to sebaceous glands and sebocytes with terms describing experimental models, 3D systems, organoids, tissue engineering, and relevant applications. The main search terms included: “sebaceous gland” OR “sebaceous glands” OR “sebocyte” OR “sebocytes”, combined with “experimental model” OR “in vitro” OR “ex vivo” OR “animal model” OR “primary sebocyte” OR “sebocyte cell line” OR “immortalized sebocyte” OR “3D culture” OR “spheroid” OR “organoid” OR “skin equivalent” OR “organotypic culture” OR “co-culture” OR “microfluidic” OR “bioengineered skin”. Additional searches combined SG- and sebocyte-related terms with specific applications, including “lipid metabolism”, “skin microbiome”, “immune cells”, “vascularization”, and “innervation”.

## 3. Animal Models

To study the biology and diseases of SGs, several animal models have been developed [31,32]. A well-known example is the asebia mouse, a classical model for investigating the pathophysiology of primary cicatricial alopecia. This model is characterized by SG atrophy resulting from a spontaneous mutation in the gene encoding stearoyl-CoA desaturase 1 (*Scd1*) [33]. Most research efforts have focused on the development of animal models for acne. However, many early acne models did not require bacterial infection to induce disease and therefore failed to fully recapitulate the human condition. To overcome this limitation, several acne-like animal models have been established in which lesions are induced by intradermal injection of *Cutibacterium acnes* (*C. acnes*) into the dorsal skin of mice. These models have been widely used to evaluate the efficacy of novel therapeutic agents. For instance, Su et al. [34] demonstrated that, in a *C. acnes*-induced mouse model, lactoferrin, a multifunctional glycoprotein abundant in body fluids, particularly colostrum, modulates sebogenesis, reduces SG lipid content, attenuates inflammation, and inhibits sebocyte proliferation while promoting apoptosis. To better mimic the coexistence of increased sebum production and inflammation, which are hallmarks of acne, more recent models combine intradermal *C. acnes* injection with topical application of human sebum or squalene, a lipid enriched in sebum of acne patients [35,36,37]. Consistent with previous findings [38], this combined approach induces characteristic inflammatory lesions, including papules and pustules, along with dermal inflammatory cell infiltration [37]. Moreover, lipid accumulation and the expression of lipogenic proteins are significantly increased (see Table 1). Using this model, Yan et al. [36] showed that the therapeutic efficacy of 5-aminolevulinic acid photodynamic therapy (ALA-PDT), a clinically established treatment for moderate-to-severe acne, is associated with rapid resolution of acneiform lesions. This effect appears to involve an initial transient exacerbation of inflammation, followed by a reduction in inflammation, sebum production, and SG size, in agreement with previous reports [39]. Additional animal-derived sebocyte models include rat preputial sebocytes [40] and hamster sebocytes [41]. In male rodents, preputial glands (PGs) resemble SGs and consist of lipid-producing sebocytes. They are frequently used as a surrogate model due to their larger size and ease of isolation [42,43]. Accordingly, PG-derived sebocytes have been widely employed in studies of sebocyte biology [40,44,45,46]. However, recent single-cell RNA sequencing analyses have highlighted important differences between SG- and PG-derived sebocytes [47]. Although both cell types are involved in lipid production, they display distinct gene expression profiles, particularly in pathways related to specialized lipid class biosynthesis. Notably, genes associated with the mevalonate and sphingolipid salvage pathways are less expressed in PG sebocytes. These findings indicate significant tissue-specific differences between sebocyte populations, suggesting that PGs may not fully recapitulate the biology of cutaneous SGs and should be used with caution.

In recent years, the use of animal models has declined due to ethical concerns, high costs, and species-specific differences in sebum composition. For example, certain lipids such as squalene may be absent or present in different proportions across species [12]. Moreover, structural and molecular differences exist in SGs, including species-specific expression of sebaceous markers; keratin 7 (K7) and mucin 1 (MUC1) are expressed in human SGs but not in murine SGs [31]. These differences may explain why SG-associated diseases vary between species, with acne being a uniquely human disease. Although rodent models, particularly those incorporating sebocyte-specific genetic manipulation [48], remain valuable, the study of human sebocytes is essential for a comprehensive understanding of SG biology, sebum function, and SG-associated diseases.

## 4. Human SGs and Primary Sebocytes

For these reasons, considerable efforts have been devoted to optimizing methods for the isolation of human SGs. In 1966, Kellum reported an early method for isolating SGs from human skin; however, viable glands could not be maintained in vitro [49]. In 1982, Karasek and Charlton briefly described the successful culture of human sebocytes from skin slices enriched in SG tissue, although no detailed study was subsequently published [50]. In 1986, Kealey et al. successfully maintained isolated human SGs ex vivo for a limited period [51]. This model was later employed to investigate several aspects of sebaceous biology, including lipogenesis [52]; the effects of sex hormones and retinoids [53]; cellular metabolism, such as aerobic glycolysis and glutaminolysis [54]; and the role of interleukins, growth factors, peroxisome proliferator-activated receptors (PPARs), farnesoid X receptor ligands, and Toll-like receptor 2 (TLR2) in sebocyte differentiation [54,55]. However, a major limitation of ex vivo SG models is the short lifespan of sebaceous glands once isolated from the skin, which restricts their use in long-term or large-scale studies [42,50]. In addition, significant inter-donor variability, partly related to age, can lead to inconsistent results.

The isolation and culture of primary human sebocytes represent an alternative approach to overcome these limitations. In 1989, Xia and colleagues described the first detailed protocol for the isolation, characterization, and maintenance of human sebocytes in vitro [56]. In this method, SGs were isolated from human pilosebaceous units, the ducts were removed, and the glandular lobules were subsequently cultured on a feeder layer of 3T3 fibroblasts (murine embryonic fibroblast cells commonly used to support epithelial cell growth). After 2–3 weeks, sebocytes could be isolated and passaged up to three times. Additional approaches include the method described by Doran et al., based on enzymatic dissociation of skin-derived cells [57], and the protocol by Fujie et al., in which sebocytes were obtained either by explant culture or enzymatic dissociation of SGs without the use of a feeder layer [58]. Both approaches yielded viable cells exhibiting characteristic sebocyte features, such as lipid droplet accumulation and the expression of differentiation markers (see Table 1). Key quality criteria for sebocytes include the absence of keratinocyte markers and the inability to form a cornified envelope. More recently, Nelson et al. demonstrated that treatment with Y-27632, a Rho-associated protein kinase (ROCK) inhibitor, enables the expansion of primary human sebocytes in vitro [59]. Nevertheless, primary human sebocyte cultures display considerable heterogeneity, making careful characterization essential prior to downstream applications. In addition, primary sebocytes exhibit a lower proliferative capacity compared with keratinocytes derived from the same donor [60] and show a strong tendency toward spontaneous differentiation in vitro [61]. Importantly, primary sebocytes also undergo incomplete terminal differentiation under conventional culture conditions, and their phenotype may progressively diverge from that of sebocytes within intact SG [61,62,63]. Thus, although primary sebocytes retain important physiological characteristics and provide a valuable model that avoids oncogene-driven immortalization, their limited proliferative capacity, spontaneous differentiation, incomplete terminal differentiation, and inter-donor heterogeneity constrain their use in long-term, large-scale, and highly reproducible experiments. These limitations have motivated the development of immortalized sebocyte cell lines, which provide a complementary experimental approach with substantially greater proliferative capacity and reproducibility.

## 5. Immortalized Human SG Cell Lines

The first immortalized human SG cell line, SZ95, was established by Zouboulis and colleagues [64]. Sebocytes were isolated from the facial skin of an 87-year-old woman and immortalized by transfection with the simian virus 40 (SV40) large T antigen. SZ95 sebocytes express characteristic sebocyte markers, accumulate cytoplasmic lipid droplets, and synthesize key sebaceous lipids (see Table 1). They also respond to known modulators of sebaceous lipogenesis, including androgens and retinoids [64,65]. Consequently, SZ95 sebocytes have become a widely used model for studying sebocyte biology and SG pathophysiology. In 2003, Thiboutot and colleagues established a second immortalized human sebocyte cell line, SEB-1, derived from the periauricular region of a 55-year-old male donor [66]. This cell line was generated using a similar SV40-mediated immortalization approach. In 2008, in collaboration with the Zouboulis group, Lo Celso et al. established another human sebocyte cell line (Seb-E6/E7) using an alternative approach based on the transfection of human papillomavirus type 16 (HPV16) *E6* and *E7* genes [67]. Building on this strategy, Barrault et al. [68] developed the SEBO662 model, which enabled the generation of a reconstructed 3D sebaceous epithelium, providing a more physiologically relevant platform for sebocyte research. More recently, Liu et al. [69] generated a human telomerase reverse transcriptase (hTERT)-immortalized sebocyte cell line of Asian origin (XL-i-20). This model retains key features of primary sebocytes, including morphology, marker expression, lipid production, and inflammatory responses, and therefore represents a valuable tool for studying SG biology across diverse populations. The major immortalized human sebocyte cell lines differ substantially in donor characteristics, immortalization strategy, degree of sebaceous differentiation, and experimental applications (Table 2).

SZ95 remains the most extensively characterized and widely used human sebocyte model, whereas SEB-1 has been particularly useful for investigating sebaceous steroidogenesis. Seb-E6/E7 and SEBO662 provide alternative HPV16 E6/E7-based models, with SEBO662 being particularly suitable for 3D sebaceous epithelial reconstruction. More recently, XL-i-20 has expanded the available models to an Asian-derived sebocyte line and has enabled the development of sebaceous gland organoids. These differences should be considered when selecting a model, as cellular responses may depend not only on the biological stimulus but also on the origin of the donor, immortalization strategy, differentiation state, and culture conditions.

Overall, immortalized sebocyte cell lines have significantly contributed to our understanding of sebocyte biology, including their roles in skin homeostasis, lipid metabolism, and inflammation. Their major advantages include long-term proliferative capacity, experimental reproducibility, and the ability to generate sufficient numbers of cells for mechanistic and large-scale studies. However, these advantages must be balanced against important biological limitations. Unlike primary sebocytes, immortalized sebocytes have been adapted for sustained proliferation through genetic manipulation. In particular, SV40 large T antigen-mediated immortalization, as used for SZ95 and SEB-1 cells, directly interferes with p53-dependent signaling [70,71,72], whereas HPV16 E6/E7-mediated immortalization of Seb-E6/E7 human sebocyte cell line affects both p53 and pRB pathways [67,73]. These alterations may influence downstream signaling networks involved in cell-cycle progression [74], autophagy [75], apoptosis [74], and lipid metabolism [76,77,78], potentially modifying cellular responses to experimental stimuli. Therefore, the preservation of sebocyte markers, lipid accumulation, or responsiveness to selected hormonal and pharmacological stimuli does not necessarily indicate complete equivalence to primary sebocytes or intact human SGs. Moreover, immortalized sebocyte lines generally exhibit incomplete sebaceous differentiation and do not fully reproduce the terminal holocrine differentiation and secretion occurring in vivo [50]. Most studies also rely on two-dimensional (2D) culture systems, which fail to reproduce the complex three-dimensional (3D) architecture, cell–cell interactions, extracellular matrix, and tissue-specific microenvironment of sebaceous glands in vivo. In conventional monolayer cultures, sebocytes grow under conditions that differ substantially from their native tissue context, limiting the physiological relevance of these models [50]. Accordingly, immortalized sebocyte lines should be considered complementary experimental models rather than complete surrogates of human SGs.

## 6. Sebocyte-Based Co-Culture and Organotypic Skin Models

Sebocytes are now recognized as key players in the crosstalk with other cell populations within the skin. Numerous studies highlight their critical role in maintaining epidermal integrity and homeostasis through interactions with multiple skin cell types. Evidence from sebocyte–skin co-culture systems indicate that sebocytes contribute to keratinocyte differentiation and epidermal homeostasis. These effects are likely mediated by growth factors and hormones produced by sebocytes, including sex steroids such as estrogens and androgens, which are known to regulate keratinocyte function and tissue homeostasis [26]. Consistently, skin cultured in the absence of sebocytes exhibits degenerative changes, underscoring their essential role in preserving epidermal integrity [27,28]. Beyond keratinocytes, sebocyte-derived factors also influence fibroblast behavior and contribute to the maintenance of epidermal melanocytes [29,30]. Increasing evidence supports a key role for sebocytes in regulating melanocyte biology and skin pigmentation. Notably, body regions enriched in sebaceous glands, such as the face, axillae, and genitalia, display higher melanocyte density and pigmentation levels [79]. Co-culture of SZ95 sebocytes with melanocytes has been shown to promote melanocyte proliferation and dendricity [29], while sebocyte-derived lipids, particularly UV-oxidized squalene, can stimulate melanocyte proliferation and melanin synthesis in organ culture models [80]. Ex vivo co-culture systems further reinforce the role of sebocytes in melanogenesis. Skin explants co-cultured with SZ95 sebocytes exhibit increased melanin content, associated with enhanced expression of stem cell factor (SCF) [30], a key regulator of melanocyte function and a mediator of hyperpigmentation disorders [81]. These models also confirm that sebocytes contribute to improved epidermal structural integrity, supporting previous findings [26]. Given that sebocytes are a major source of sebum-associated proteins [82], the increased levels of p53 and melanogenic growth factors detected in sebum from melasma lesional compared to non-lesional skin further support an active role of sebaceous glands in regulating pigmentation. Overall, these findings indicate that sebocytes are integral components of a complex cell–cell communication network that coordinates skin homeostasis, pigmentation, and responses to environmental stress.

To investigate these interactions in a physiologically relevant tissue context, sebocytes have also been incorporated into ex vivo skin models. Skin explant models are derived from full-thickness or split-thickness human skin obtained during surgical procedures or biopsies and are maintained ex vivo in appropriate culture media to preserve their native architecture [83]. In parallel, human skin equivalents (HSEs) are generated by reconstructing skin layers through the sequential assembly of isolated cell populations, resulting in a living and interactive tissue-like structure [84]. Skin explants offer several advantages, including ease of procurement, preservation of all resident skin cell types and appendages, and maintenance of the native lipid barrier composition. HSEs, in contrast, provide greater experimental reproducibility and flexibility [85]. Compared with conventional 2D cultures, 3D culture systems more accurately recapitulate tissue architecture and cellular functions, thereby better reflecting in vivo conditions and enabling more physiologically relevant cell–cell and cell–matrix interactions [86,87]. In addition, 3D cultures exhibit enhanced structural stability and resilience to external stress, properties that are not achievable in traditional 2D monolayer systems [88,89]. In a 3D system, cells establish complex interactions with neighboring cells and extracellular matrix (ECM) components, further contributing to tissue-like organization [90]. Consequently, 3D cultures systems represent an advanced platform that bridges the gap between conventional in vitro models and in vivo animal studies [91]. To better recapitulate in vivo skin complexity, various cell types have been incorporated into HSEs, including melanocytes, Langerhans cells, endothelial cells, and sweat gland cells [92,93,94,95]. Similarly, pilosebaceous units have been integrated into organotypic culture systems [96], and skin organ cultures have been used to induce hair follicle formation [97]. To investigate the interactions between SGs and other skin compartments, Nikolakis et al. developed the 3D-SeboSkin model in 2015 [26] in which skin explants are maintained in direct contact with SZ95 sebocytes. This sebocyte-based co-culture system demonstrated improved epidermal structural integrity. After six days in culture, an increased proportion of proliferating basal keratinocytes and reduced apoptosis of differentiating epidermal cells were observed, as assessed by Ki-67 and TUNEL staining, respectively. In parallel, SZ95 sebocytes maintained normal differentiation and lipid accumulation. Notably, interleukin-6 expression was reduced in co-cultures compared with skin explants cultured alone. These findings support the existence of beneficial crosstalk between sebocytes and the surrounding skin microenvironment and highlight the relevance of incorporating SG components into 3D skin models. Accordingly, the 3D-SeboSkin model has been successfully applied in ex vivo studies of inflammatory skin disorders, including hidradenitis suppurativa (acne inversa), as well as in investigations of pathogen-associated molecular patterns [27,28].

Despite these advances, skin explant and 3D skin equivalent models still suffer from several shortcomings. Skin explants undergo progressive cellular degeneration during ex vivo culture, whereas reconstructed 3D systems often rely on simplified cellular compositions, and variability in culture protocols may affect reproducibility and data interpretation. Moreover, even advanced 3D systems fail to fully recapitulate key aspects of skin physiology, such as vascularization, immune complexity, and innervation. While ex vivo reinnervation approaches may partially address these limitations [98], constraints related to nutrient diffusion and oxygen availability limit the size of 3D constructs and may influence their responses to external and neurotrophic stimuli.

## 7. Sebocyte Spheroids

Recent advances in 3D culture technologies have improved the applicability of SG cell lines for generating 3D SG-like structures and modeling SG biology and disease. In this context, we adopted an operational classification to distinguish different types of 3D aggregates based on the cellular population used and the degree of structural complexity achieved. Within this framework, we use the term “sebocyte spheroids” primarily for 3D aggregates generated from immortalized sebocyte cell lines, which exhibit a relatively simple architecture and limited capacity for self-organization compared with more complex multicellular models. Although these systems can reproduce selected morphological and functional features of SGs, they do not fully recapitulate the multicellular organization and developmental complexity of native glands. When cultured under 3D conditions, SZ95 sebocytes form spheroid structures surrounded by basement membrane components. These spheroids display increased size and structural complexity upon exposure to prostaglandin E2, along with activation of the canonical Wnt signaling pathway compared with control conditions. These changes are associated with enhanced cell viability, proliferation, mitochondrial activity, and lipid synthesis [99,100]. Despite these advantages, this method presents several limitations, including long production times, high costs, and variability in spheroid size and shape. For instance, traditional techniques such as hanging drop and liquid overlay methods typically require at least 48 h to generate mature spheroids [101], and often produce structures with heterogeneous morphology, leading to reduced reproducibility. Moreover, prolonged culture times may negatively affect cell viability within spheroids. Recent improvements in 3D culture methodologies have significantly reduced spheroid formation time. In particular, the inertial focusing effect-based hanging drop method accelerates cell aggregation by inducing cell accumulation at the base of rotating droplets, thereby promoting rapid self-assembly [102]. This approach enables the formation of uniform and reproducible spheroids within 12 h, while reducing costs and improving scalability. Using this technique, Tang et al. [103] generated sebocyte spheroids that closely resemble in vivo SG architecture, with controllable size achieved by adjusting the number of seeded cells. To mimic human SG dimensions (approximately 100–250 µm), around 1 × 10^3^ SZ95 cells were used. These sebocyte spheroids remained structurally stable for at least five days. After 24 h, they exhibited the expression of E-cadherin and laminin, indicating the formation of compact structures with ECM components. Furthermore, increased expression of Blimp-1, a marker of both SG progenitors and terminal sebocyte differentiation (see Table 1), was observed compared with monolayer cultures, suggesting enhanced sebocyte differentiation in the 3D system. More recently, Liu et al. [69] established human SG spheroids using high-concentration matrigel combined with immortalized XL-i-20 sebocytes. These 3D structures expressed key sebocyte markers (see Table 1) as well as PLET1, a marker associated with sebaceous progenitor cells, and exhibited functional responses to 5-aminolevulinic acid photodynamic therapy (ALA-PDT), supporting their relevance as an in vitro model for research on SGs.

A major challenge in acne research is that conventional in vitro systems fail to reproduce the complex 3D microenvironment of the skin, including inflammatory cues and interactions between sebocytes and immune cells. To address this limitation, Tang et al. [104] developed a 3D co-culture model consisting of sebocyte spheroids and macrophages using an inertial focusing approach. This system recapitulates a controllable inflammatory microenvironment resembling acne pathogenesis and allows for separate analysis of sebocytes and immune cells. Their findings indicate that pro-inflammatory macrophages promote lipid accumulation and oxidative stress in sebocytes, while sebocyte-derived signals induce macrophage polarization toward a pro-inflammatory M1 phenotype. These models represent a transition toward more physiologically relevant systems, paving the way for fully organotypic and patient-specific SG models.

## 8. SG Organoids

In accordance with the operational definition outlined above, based primarily on cell source and organizational complexity, we use the term “SG organoids” to refer to 3D structures generated from primary sebocytes or stem/progenitor cells that exhibit greater capacity for self-organization and can reproduce multiple cellular, structural, and functional features of SGs. Advances in skin organoid technology have led to the development of multicellular 3D models that closely recapitulate human skin architecture [105,106]. These systems have evolved into complex and physiologically relevant structures and are increasingly recognized as powerful alternatives to conventional culture models, providing essential tools for studying sebocyte differentiation and lipid production. Their intrinsic plasticity enables the generation of both physiological and pathological models, offering valuable insights into skin development and dynamic tissue remodeling. Recent progress has enabled the establishment of robust protocols for the generation of SG organoids that reproduce key morphological, structural, and functional features of their in vivo counterparts [107]. Engineering strategies allow precise control over cell behavior, microenvironmental conditions, and cell–matrix interactions, thereby enhancing the physiological relevance of these models. SG organoid engineering integrates principles from developmental biology, stem cell research, and bioengineering. The organization and function of SG organoids are tightly regulated by interactions between stem cells and their niche. Accordingly, successful organoid generation depends on both the availability of appropriate stem cell sources and the reconstruction of a microenvironment that closely mimics the native SG niche.

### 8.1. Stem Cell Sources and Generation of SG Organoids

SG organoids can be derived from two principal stem cell sources: adult stem cells (ASCs) and pluripotent stem cells (PSCs). These cells possess the capacity to self-organize into structures that recapitulate key features of sebaceous glands [108]. ASC-derived SG organoids have been generated by Feldman et al. [109] from mouse Blimp1-positive SG stem cells [109], consistent with earlier findings identifying Blimp1 as a marker of SG progenitor cells [110]. Following isolation and expansion, these cells are embedded in matrigel and cultured in media supplemented with morphogenetic factors such as FGF and EGF. Within 7–12 days, SG-like structures form, characterized by an outer proliferative layer and an inner compartment of differentiated, lipid-producing sebocytes. These organoids exhibit asymmetric cell division in the outer layer cells and produce SG-specific lipids, indicating functional similarity to in vivo glands. Notably, c-Myc was identified as an important regulator of both sebocyte proliferation and differentiation in this system. Moreover, Boonekamp et al. [111] demonstrated the long-term expansion and differentiation of adult murine epidermal stem cells in 3D organoid cultures, providing further evidence for the potential of adult stem/progenitor cells as a source for skin organoid models. PSC-derived models have further expanded the potential of SG organoids. For example, human skin organoids containing both hair follicles (HFs) and SGs have been generated from the WA25 human embryonic stem cell line [112,113]. In this system, single cells are first separated to generate uniform aggregates and then induced toward an ectodermal lineage using a differentiation medium containing Matrigel, BMP4, and the TGF-β inhibitor SB431542. Subsequent patterning toward surface ectoderm is achieved using BMP inhibitors and FGF. After approximately 120 days, complex skin organoids containing hair follicles and SGs are formed. These models provide a powerful platform to investigate genetic determinants, signaling pathways, and molecular mechanisms regulating SG development and function. A recent study described “sebonoids”, i.e., primary human sebocyte-derived 3D organoids generated from primary human sebocytes, and provided a standardized protocol for their generation and application, highlighting their potential for studying sebaceous gland growth and differentiation and for modeling SG-associated diseases [114]. Compared with immortalized sebocyte models, these systems may better preserve progenitor-like characteristics and tissue-specific differentiation, although their reproducibility and long-term maturation still require further validation.

Despite these advances, important challenges remain in current SG organoid systems, especially regarding incomplete cellular maturation [115]. In this context, the incorporation of additional physiological components, such as vascular, neural, and immune elements, has emerged as a key strategy to enhance organoid complexity and functionality [116].

### 8.2. Vascularized and Innervated SG Organoids

In native tissue, SGs are surrounded by a dense vascular network that supplies oxygen, nutrients, signaling molecules, and hematopoietic cells, all of which are essential for gland development and homeostasis [117]. However, the lack of vascularization remains a major limitation in conventional organoid systems, restricting tissue size, structural organization, and functional maturation. Current approaches to vascularization rely on the incorporation of endothelial progenitor cells (EPCs), often derived from PSCs, combined with advanced bioengineering strategies and suitable extracellular matrix (ECM) scaffolds [117]. Microfluidic platforms and 3D bioprinting technologies enable the formation of perfusable vascular-like networks by guiding endothelial cell self-organization within hydrogels [118,119]. ECM composition and biophysical properties, including stiffness, degradability, and integrin-binding capacity, play a critical role in stabilizing these networks [117]. Although significant progress has been made, maintaining long-term stability and functionality of microvasculature within SG organoids remains a major technical challenge.

Innervated SG organoids are another emerging frontier. Innervation plays a crucial role in regulating organ development and function, yet its contribution to SG biology remains incompletely understood [7]. While classical SGs associated with HFs may be indirectly influenced by neural inputs through the follicular niche, specialized SGs independent of HFs (meibomian and preputial glands) have been shown to possess dense nerve fiber networks, suggesting a direct regulatory role [120]. Recent strategies to incorporate neural components into organoids include co-culture systems integrating PSC-derived neural crest cells with epithelial organoids, as well as 3D bioprinting approaches that enable spatially controlled assembly of neural and epithelial compartments [121,122,123]. A key challenge lies in selecting appropriate neural crest-derived populations to achieve physiologically relevant innervation patterns in SG organoids.

### 8.3. Interplay Between SGs, Immune Cells, and Skin Microbiota in 3D Skin Models Containing SG

Another key aspect is the dynamic interplay between SGs, immune cells, and skin microbiota. SGs contribute to skin barrier function through sebum production, which shapes microbial composition and modulates immune responses, while immune cells in turn regulate SG activity, establishing a bidirectional crosstalk essential for tissue homeostasis [124,125,126]. To investigate these interactions, co-culture systems incorporating various immune cell types have been developed, including air–liquid interface models that enable direct epithelial–immune and host–microbe interactions [127,128,129]. However, maintaining the viability and functionality of multiple cell types within the same culture system remains a major challenge due to their distinct growth factors requirements. The incorporation of live microbiota into 3D skin models and skin organoids introduces additional technical challenges. A major limitation is the need to simultaneously preserve host tissue viability and differentiation while providing suitable conditions for microbial survival and colonization. Conventional culture media are primarily optimized for mammalian cells and may not reproduce the nutrient availability, oxygen gradients, and physicochemical conditions of the skin surface, potentially altering microbial growth, metabolism, and community composition. Conversely, uncontrolled microbial proliferation may compromise tissue viability through nutrient depletion and accumulation of microbial metabolites. Air–liquid interface systems can facilitate microbial exposure at the epidermal surface while preserving tissue differentiation but maintaining stable and physiologically representative microbial communities remains challenging. Most current models rely on individual species or simplified microbial consortia, which facilitate experimental control but do not reproduce the complexity, interspecies interactions, and metabolic heterogeneity of the native skin microbiota. Moreover, microbial colonization must remain spatially restricted to the tissue surface without compromising epithelial barrier integrity or causing uncontrolled invasion. Emerging approaches, including air–liquid interface cultures, microfluidic platforms, oxygen-controlled systems, and engineered extracellular matrices, may help overcome these limitations by providing more appropriate spatial and physicochemical conditions for host–microbiota co-culture. Nevertheless, achieving reproducible and long-term colonization by diverse microbial communities while preserving tissue viability and organoid functionality remains a major challenge for the development of physiologically relevant SG-containing skin models [130,131,132].

### 8.4. Multi-Omics Integration and Functional Validation

Recent efforts to improve the robustness and physiological relevance of SG organoids have increasingly relied on bioengineering approaches, including fine-tuning of the microenvironment, development of advanced biomaterials, and modeling the effects of hormones, genetic alterations, and environmental stressors [69]. In parallel, the integration of multi-omics approaches with 3D skin models and skin organoids provides complementary molecular and functional readouts of sebocyte maturation. Single-cell RNA sequencing (scRNA-seq) can resolve cellular heterogeneity within organoids and identify distinct sebocyte states and differentiation trajectories, whereas spatial transcriptomics preserves tissue architecture and enables the localization of stage-specific transcriptional programs within the organoid. These transcriptomic approaches can be further complemented by lipidomic profiling, which provides a direct functional assessment of sebaceous differentiation by determining whether organoids produce lipid species and lipid profiles comparable to those of native SGs. For example, Blimp1-positive cell-derived SG organoids have been characterized by LC-MS lipidomics and shown to produce a lipid signature resembling that of native SGs, demonstrating how molecular profiling can be integrated with phenotypic and functional analyses to validate organoid fidelity [109]. More recently, integrated single-cell and spatial transcriptomic analyses of human SGs have identified distinct stages of sebocyte differentiation and their associated gene expression programs, providing a reference framework against which organoid-derived cellular states can be compared [9,133]. The integration of organoid biology with engineering and multi-omics approaches therefore enables a multidimensional assessment of SG models, linking cellular identity and differentiation (scRNA-seq), spatial organization (spatial transcriptomics), and functional lipid production (lipidomics). Such an integrated framework may help identify maturation defects, aberrant signaling pathways, or metabolic alterations that are not readily detectable through conventional morphological or single-marker analyses. Consequently, SG organoids have emerged as a highly promising platform for fundamental research, disease modeling, drug discovery, and regenerative medicine. These models are increasingly applied to investigate skin appendage development, including SGs and HFs, as well as to support personalized medicine and functional skin regeneration [69].

## 9. Bioengineered, Microfluidic, and Standardized SG-Containing Models

Beyond conventional organoid approaches, recent advances in bioengineering and microfluidic technologies have enabled the development of more modular, scalable, and spatially controlled SG models. SebaSpheres represent a recent example of a microfluidics-based platform generating uniform 3D SG-like structures that accumulate lipids and express the sebocyte differentiation marker Blimp-1. These structures can subsequently be integrated with fibroblasts and keratinocytes to generate a multicellular SG-containing skin model, illustrating the potential of microfluidic approaches to generate standardized and modular SG-containing systems [134]. Similarly, standardized 3D/ex vivo platforms have been developed to investigate SG physiology and host–microbiota interactions. For example, a human 3D ex vivo SG model has been established and successfully colonized with Cutibacterium acnes and Staphylococcus epidermidis, providing a platform for investigating sebaceous responses to microbial colonization and acne-associated dysbiosis [135]. Commercially available engineered platforms, such as the 3D SEBOSkin model, further illustrate the ongoing translation of SG tissue engineering toward standardized and reproducible research systems. From a translational perspective, the scalability, reproducibility, throughput, and resource requirements of these platforms are also important considerations for their broader adoption, particularly when compared with conventional 2D and more complex 3D systems. However, independent validation and benchmarking against native human SGs remain essential to establish the physiological relevance and reproducibility of these models.

## 10. Future Perspectives

Despite significant progress in the development of experimental models for SG research over the past decades, several key questions remain unresolved. Addressing these challenges will be essential to fully understand SG biology and its role in skin homeostasis and disease [50]. Future research should focus on elucidating how alterations in sebum production and composition influence the complex interplay between the immune system, the skin microbiome, and SG function, including their impact on sebocyte activity and skin inflammation. In addition, a deeper understanding of hair follicle development and its integration with SG biology remains a critical objective. To prioritize these challenges, future efforts can be broadly organized into three complementary stages: short-term standardization of existing models, mid-term integration of increasingly complex microenvironments, and long-term development of patient-derived and clinically predictive platforms.

In the short-term, the priority should be the systematic validation and standardization of existing SG models. Recent technological advances have provided new opportunities to overcome several limitations of current SG models. However, progress should move beyond the generation of increasingly complex models toward their rigorous comparison with native human SGs. Single-cell and spatial transcriptomics, lipidomics, proteomics, and functional assays will be essential to determine whether engineered models reproduce the molecular, metabolic, and structural characteristics of human glands [9,109,133]. Establishing common quality control criteria and quantitative benchmarks for sebocyte identity, differentiation, lipid production, tissue architecture, and response to physiological or pathological stimuli will also facilitate reproducibility and comparison between laboratories. These benchmarks should provide a common framework for evaluating model fidelity, reproducibility, and biological performance before more complex systems are adopted for translational applications.

In the mid-term, the focus should shift toward integrating the multiple cellular and environmental components that contribute to the SG microenvironment. Patient-derived SG organoids, bioengineered SG-containing 3D systems, microfluidic platforms, and organ-on-a-chip technologies can provide increasingly precise control over cellular composition, extracellular matrix properties, biochemical gradients, and tissue architecture. The integration of vascular, immune, neural, and microbial components into these systems may further improve their ability to reproduce the dynamic SG microenvironment. In parallel, advances in 3D bioprinting and biomimetic scaffolds may enable the spatial organization of sebocytes, keratinocytes, fibroblasts, endothelial cells, and other relevant cell populations, thereby facilitating the generation of more complex and reproducible SG-containing tissues [116,117,118,119,136,137]. An important objective at this stage will be to determine which components are necessary to reproduce specific aspects of SG physiology and disease, rather than simply increasing model complexity. This approach should facilitate the investigation of how alterations in sebum production and composition affect the interactions among sebocytes, immune cells, microbial communities, and other skin compartments.

In the long-term, these advances may enable the development of patient-derived and clinically predictive SG platforms. More physiologically relevant human SG models could reduce reliance on animal models and provide improved platforms for investigating acne, hidradenitis suppurativa, seborrhoea, pigmentation disorders, and other SG-associated diseases. Patient-specific organoids and ex vivo systems may ultimately enable the testing of therapeutic responses in a personalized context, while standardized 3D and microfluidic platforms could support medium- to high-throughput drug screening and the identification of novel sebocyte- or lipid-targeting compounds. The incorporation of host–microbiota interactions may further improve the predictive value of these models for therapies targeting the complex relationship between sebaceous lipids, microbial communities, and inflammation. For these applications to become clinically relevant, however, rigorous benchmarking against human tissue, assessment of inter-individual variability, scalability, long-term stability, and evidence that in vitro responses can predict clinical outcomes will be required.

Ultimately, the progressive integration of advanced experimental models, increasing multicellular and microenvironmental complexity, patient-derived systems with multi-omics, spatial profiling, quantitative imaging, and computational approaches is likely to shift SG research from descriptive characterization toward predictive and mechanism-based modeling. Combining cellular and molecular readouts with patient-derived clinical and phenotypic information may enable the identification of disease-specific sebocyte states, biomarkers of therapeutic response, and novel molecular targets. Thus, rather than pursuing model complexity as an end point in itself, future SG research should prioritize a stepwise transition from validated and standardized systems to integrated models and, in the long-term, patient-specific platforms with demonstrated clinical predictive value.

## Figures and Tables

**Table 1:** Key molecular and phenotypic parameters used for the characterization and validation of experimental models of human sebaceous gland biology.

Parameter/Marker	Biological Relevance	Main Aspect of SG Biology Assessed
**KRT7 (CK7)**	Sebocyte-associated epithelial/lineage marker	Sebocyte identity and differentiation
**EMA**	Sebaceous epithelial/differentiation-associated marker	Sebocyte identity and differentiation
**MUC1**	Sebaceous epithelial marker associated with sebocyte differentiation	Sebocyte identity and differentiation
**Blimp-1 (PRDM1)**	Transcriptional regulator associated with sebocyte differentiation and terminal maturation	Sebocyte differentiation and terminal maturation
**PPARγ**	Transcriptional regulator of sebocyte differentiation and lipid metabolism	Sebocyte differentiation and lipogenesis
**SREBP1**	Central regulator of lipogenic gene expression	Lipid synthesis and sebocyte maturation
**FASN**	Enzyme involved in de novo fatty-acid synthesis	Lipogenic activity
**Perilipin**	Lipid-droplet-associated protein	Intracellular lipid accumulation and sebocyte maturation
**Sebaceous lipid production**	Accumulation/synthesis of characteristic sebaceous lipids, including triglycerides, wax esters, squalene, and free fatty acids	Functional sebocyte differentiation and lipogenesis
**Sebocyte morphology and lipid droplets**	Progressive enlargement and cytoplasmic lipid accumulation during differentiation	Phenotypic differentiation and maturation
**Holocrine differentiation**	Terminal sebocyte differentiation followed by cellular disintegration and secretion	Functional maturation and physiological relevance

**Table 2:** Characteristics and applications of immortalized sebocyte cell lines.

Cell Line	Donor/Origin	Immortalization	Main Characteristics	Main Applications	Key Limitations
**SZ95**	Facial skin; 87-year-old woman	SV40 large T antigen	K7/EMA expression; lipid droplet accumulation; production of sebaceous lipids; responds to androgens and retinoids	Sebocyte proliferation and differentiation, lipogenesis, inflammation, hormonal regulation, pharmacological studies	SV40-mediated p53 disruption; limited mature sebocyte phenotype; predominantly 2D culture
**SEB-1**	Periauricular skin; 55-year-old man	SV40 large T antigen	Sebocyte-like morphology; lipid accumulation; steroidogenic and androgen-related responses	Steroidogenesis, androgen metabolism, sebocyte lipid biology	SV40-associated alterations in cellular signaling; less extensively characterized than SZ95
**Seb-E6/E7**	Adult facial skin	HPV16 E6/E7	Sebocyte-like morphology and differentiation; alternative immortalization strategy to SV40	Sebocyte differentiation and molecular regulation	HPV16 E6/E7 interference with p53/pRB pathways; limited characterization
**SEBO662**	Sebaceous scalp nevus; 1-year-old male	HPV16 E6/E7	Enhanced sebaceous differentiation and lipid production in 3D culture	3D sebaceous epithelial reconstruction; differentiation studies	Limited differentiation in 2D culture; phenotype strongly dependent on culture conditions
**XL-i-20**	Facial skin; 43-year-old Chinese woman	hTERT	Sebocyte-like morphology, lipid production and inflammatory responses; long-term proliferative capacity	SG biology, lipid metabolism, inflammation, organoid models	Less extensively validated; immortalization may alter signaling and differentiation.

## Data Availability

No new data were created or analyzed in this study. Data sharing is not applicable to this article.
